# Pharmacokinetics of lamivudine, abacavir and zidovudine administered twice daily as syrups versus scored tablets in HIV-1-infected Ugandan children

**DOI:** 10.1186/1758-2652-13-S4-P176

**Published:** 2010-11-08

**Authors:** PK Gitta, L Kendall, V Musiime, K Adkison, AA Kekitiinwa, A Ferrier, O Opilo, Y Lou, MF Bwakura-Dangarembi, P Nahirya-Ntege, S Bakeera-Kitaka, M Ssenyonga, W Snowden, D Burger, AS Walker, D Gibb

**Affiliations:** 1 Paediatric Infectious Diseases Clinic (Baylor-Uganda at Mulago Hospital), P.O.Box 72052, Kampala, Uganda; 2 MRC Clinical Trials Unit, London, UK, London, UK; 3 Joint Clinical Research Centre, Kampala, Uganda; 4 GlaxoSmithKline, London, London, UK; 5 University of Zimbabwe Medical School, Harare, Zimbabwe, Harare, Zimbabwe; 6 MRC/UVRI Uganda Research Unit on AIDS, Entebbe, Uganda, Entebbe, Uganda; 7 Radboud University Nijmegen Medical Centre, the Netherlands, Nijmegen, Netherlands

## Purpose of the study

Currently there is an effort to develop simple, more convenient antiretroviral regimens for children to reduce costs and promote adherence. We report the plasma pharmacokinetics (PK) of lamivudine (3TC), abacavir (ABC) and zidovudine (ZDV) taken twice daily as syrups versus scored tablets in HIV-1 infected Ugandan children.

## Methods

Eligible children from 2 Ugandan centres in the ARROW trial, weighing 12-15kg, had taken ZDV, 3TC and ABC syrups twice daily for at least 24 weeks, and were ready to switch from syrups to scored tablets. Children were expected to remain in the 12-15kg weight band (i.e. same dosing band) for the next 4 weeks. Those with illnesses affecting PK (e.g., severe diarrhea, vomiting) were ineligible; children who missed any dose in the 3 days before sampling were excluded. Blood samples were collected at 0, 1, 2, 4, 6, 8 and 12 hours after the child’s last morning dose on syrups prior to switching to scored tablets of Combivir (ZDV+3TC) and ABC, and then repeated 4 weeks later. Adjusted Geometric Mean Ratios (aGMR) were calculated to compare plasma area under the curve (AUC0-12) and peak concentrations (Cmax) between tablets versus syrup.

## Results

19 eligible children (6 boys) were enrolled with median age of 3 (range 1.8-4) years. Following WHO tables, actual doses increased by 25% as children switched from syrups to scored tablets within the 12-15kg weight-band, and so PK parameters were normalised to the tablet dose. For ZDV and ABC, dose-normalised tablet AUC0-12 and Cmax were equivalent to syrup, but dose-normalised 3TC exposure was ~55% higher on tablets.

Actual 3TC exposure on 75mg tablet dose (AUC0-12 (%CV) 8.2 (20%) h.mg/L) was higher than expected compared to previous paediatric studies, and lower than expected for 60mg syrup dose (4.2 (36%) h.mg/L). There was no evidence of dosing or bioanalytical errors, or problems with administration (vomiting), or with integrity of the product batches, such as degradation.

## Conclusions

Although ZDV and ABC syrups and tablets gave equivalent exposures, we found higher plasma exposure from twice daily 3TC scored tablets compared to syrups. Further studies to understand the underlying mechanism for differing 3TC exposures from solution and tablets in the target population of HIV-infected children are needed.

